# Association of SDF1 and MMP12 with Atherosclerosis and Inflammation: Clinical and Experimental Study

**DOI:** 10.3390/life11050414

**Published:** 2021-05-01

**Authors:** María Marcos-Jubilar, Josune Orbe, Carmen Roncal, Florencio J. D. Machado, José Antonio Rodriguez, Alejandro Fernández-Montero, Inmaculada Colina, Raquel Rodil, Juan C. Pastrana, José A. Páramo

**Affiliations:** 1 Haematology Service, Clínica Universidad de Navarra, 31008 Pamplona, Spain; 2 Laboratory of Atherothrombosis, Program of Cardiovascular Diseases, Cima Universidad de Navarra, Instituto de Investigación Sanitaria de Navarra, 31008 Pamplona, Spain; josuneor@unav.es (J.O.); croncalm@unav.es (C.R.); fflorenciom@alumni.unav.es (F.J.D.M.); josean@unav.es (J.A.R.); 3 CIBERCV, Instituto de Salud Carlos III, 28029 Madrid, Spain; 4 Preventive Medicine, Clínica Universidad de Navarra, 31008 Pamplona, Spain; afmontero@unav.es; 5 Internal Medicine Department, Clínica Universidad de Navarra, 31008 Pamplona, Spain; icolina@unav.es (I.C.); jpastranad@unav.es (J.C.P.); 6 Internal Medicine Department, Complejo Hospitalario de Navarra, 31008 Pamplona, Spain; raklrf@unav.es

**Keywords:** SDF1/CXCR4, MMP-12, atherosclerosis, inflammation, multimarker approach, cardiovascular risk

## Abstract

BACKGROUND: Atherosclerosis is the main etiology of cardiovascular diseases (CVD), associated to systemic inflammation. Matrix metalloproteinases (MMPs) are related to atherosclerosis progression through the SDF1/CXCR4 axis promoting macrophages recruitment within the vascular wall. The goal was to assess new circulatory inflammatory markers in relation to atherosclerosis. METHODS: Measurement of SDF1, MMP12 and CRP in blood samples of 298 prospective patients with cardiovascular risk. To explore atherosclerosis progression, CXCR4/SDF1 axis and MMP12 expression were determined by RT-qPCR and by immunohistochemistry in the aorta of accelerated and delayed atherosclerosis mice models (Apoe-/- and Apoe-/-Mmp10-/-). RESULTS: SDF1, MMP12 and CRP were elevated in patients with clinical atherosclerosis, but after controlling by confounding factors, only SDF1 and CRP remained increased. Having high levels of both biomarkers showed 2.8-fold increased risk of presenting clinical atherosclerosis (*p =* 0.022). Patients with elevated SDF1, MMP12 and CRP showed increased risk of death in follow-up (HR = 3.2, 95%CI: 1.5–7.0, *p =* 0.004). Gene and protein expression of CXCR4 and MMP12 were increased in aortas from Apoe-/- mice. CONCLUSIONS: The combination of high circulating SDF1, MMP12 and CRP identified patients with particular inflammatory cardiovascular risk and increased mortality. SDF1/CXCR4 axis and MMP12 involvement in atherosclerosis development suggests that they could be possible atherosclerotic targets.

## 1. Introduction

In 2018, around 18.6 million deaths were attributed to cardiovascular disease (CVD) globally, representing the main cause of mortality around the world [1]. Atherosclerosis, a chronic inflammatory disease of the vessel wall, plays a main role in the pathogenesis of CVD [2]. From its earliest asymptomatic phase up to the late clinical stages, atherosclerosis promotes leukocyte activation, cytokine release, and infiltration of leukocytes, mainly macrophages, into the arterial wall. Indeed, recent results from the CANTOS trial have identified inflammation as an important and independent factor in CVD. Specifically, an IL-1ß inhibitor with anti-inflammatory effects, was associated with significantly lower rates of recurrent cardiovascular events [3], providing a strong evidence to support the inflammation hypothesis.

Collected data from the general population and clinical cohorts have demonstrated a strong association of some inflammatory markers, such as C-reactive protein (CRP) and CVD [4,5]. Nowadays, new systemic and imaging biomarkers are being studied as surrogate markers of cardiovascular (CV) risk and CVD [6,7]. Among them, stromal cell derived factor-1 (SDF1), located in smooth muscle cells (SMCs), endothelial cells and macrophages and present in atherosclerotic plaques, has been described as an important player in the monocytes arrest in vascular lesion and in platelet activation [8]. SDF1 and its receptor (CXCR4) are implicated in the activation of intracellular signaling pathways that promotes angiogenesis, vascular inflammatory response and neointimal hyperplasia [9]. Moreover, SDF1 has been recently identified as a causal mediator in coronary artery disease (CAD) [10,11]. The blockage of the CXCR4/SDF1 axis decreases the expression of proteolytic mediators such as MMP-12 [12], a metalloelastase secreted by macrophages and platelets, with a key role in leukocytes recruitment [13,14]. Furthermore, MMP12 has been proposed as a potential biomarker of CVD in patients with type 2 diabetes [15], while its deficiency reduced atherosclerosis development and macrophage infiltration in murine models [16].

Finally, our group has recently demonstrated a causal role of matrix metalloproteinase-10 (MMP-10) in atherosclerosis progression, macrophage infiltration and plaque calcification, using a double knock-out (2KO) mouse model (Apoe-/-Mmp10-/-). In addition, MMP-10 expression is induced by CRP in human endothelial cells [17] and both parameters positively correlated with subclinical atherosclerosis in asymptomatic subjects with CV risk factors [18]. Besides, MMP-10 activity modulates CXCR4/SDF1 signaling in skeletal muscle regeneration after experimental vascular ischemia [19].

The purpose of this study was to evaluate prospectively the circulating levels of MMP12 and SDF1 in a cohort of patients with clinical and subclinical atherosclerosis and its role as biomarkers of high risk of CVD and worse outcome. Moreover, we confirmed the association of these proteins in atherosclerosis development by assessing their expression in aortas of murine models with various degrees of atherosclerosis.

## 2. Materials and Methods

### 2.1. Patients

We performed a prospective study including 298 consecutive patients who presented more than one CV risk factor with clinical or subclinical atherosclerosis evaluated in two tertiary hospitals in Navarra (Spain), between April 2017 and June 2019. Subjects were screened with a detailed medical history, physical examination and biochemical profile. All patients underwent routine blood tests and vascular echography of carotid, abdominal aorta and femoral territories. The inclusion criteria were: (1) age > 45 years-old and (2) presence of at least two cardiovascular risk factors: hypertension (blood pressure >140/90 mmHg) or treatment with anti-hypertensive medications; diabetes mellitus (DM) or antidiabetic use, dyslipidaemia [total cholesterol >2 00 mg/dL, low-density lipoprotein cholesterol (LDL-C) > 130 mg/dL or non-HDL-C > 160 mg/dL, hypertriglyceridemia (>150 mg/dL) or on cholesterol lowering drugs]; body mass index (BMI) > 30 Kg/m^2^ or abdominal perimeter (>80 cm female or 94 cm male) and/or current smoker. Exclusion criteria were active cancer, inflammation or infection and use of nonsteroidal anti-inflammatory drugs (NSAIDs) or steroids two weeks before blood sampling.

We calculated the PREDIMED (PREvencion con DIeta MEDiterranea) score, which is a 14-item tool that reflects increasing adherence to Mediterranean diet [20].

Those patients who presented CAD, stroke or transient ischemic attack (TIA), peripheral artery disease (PAD) or nephropathy were considered as suffering clinical atherosclerosis manifestations.

Medical history of patients was reviewed in February 2021 to assess the presence of recurrence or new cardiovascular events and mortality.

Samples and data from studied patients were provided by the Biobank of the University of Navarra and processed following standard operating procedures approved by the Ethical and Scientific Committees. The Ethics Review board of the Clinica Universidad de Navarra approved the protocol (2017/240) and the study was conducted in accordance with the declaration of Helsinki. Written informed consent was obtained from each patient.

#### 2.1.1. Vascular Imaging

In order to analyze the presence of atherosclerotic plaques, an expert radiologist manually traced a line on frozen B-mode images between the far wall of the lumen-intima and the media-adventitia interfaces. All subjects were examined in the supine position. A linear array transducer (frequency of 4–9 MHz) was employed, using high-resolution B-mode system (Siemens ACUSON S2000 and S3000 devices, Siemens Healthcare, Duisburg Germany). Gain settings were optimally adjusted to facilitate edge detection.

#### 2.1.2. Blood Samples and Biochemical Analysis

Fasting serum and plasma samples were collected by venipuncture, centrifuged (20 min at 1200 g twice) and stored at −80 °C until analysis. Serum total cholesterol, high-density lipoprotein cholesterol (HDL-C), triglycerides and glucose were measured by standard laboratory techniques with Cobas 8000 (Roche Diagnostic). LDL-C was estimated using the Friedewald equation. Non-HDL cholesterol was calculated by Total Cholesterol—HDL-C. We evaluated high-sensitivity (hs)-CRP by immunoassay (Immulyte; Diagnostic Product Corporation, Los Angeles, CA, USA).

Inflammatory biomarkers, SDF1 (SDF1; Quantikine, R&D Systems, Abingdon, UK) and MMP12 (MMP12; LSBIO, lifespan Bioscience, SEA, USA), were determined by ELISA following the manufacturer instructions. Double centrifuged plasma EDTA samples were undiluted or diluted 1:300 for SDF1 and MMP12 measures, respectively. The mean coefficients of variance for intra- and inter-assay variation were 3.6% and 10.3% for SDF1 and <4.7% and <7.3% for MMP12. The detection limits were 18 pg/mL for SDF1 and 0.156 ng/mL for MMP12.

### 2.2. Experimental Mouse Model

Aorta sections were obtained from Apoe-/- mice which develop spontaneous atherosclerosis and from delayed atherosclerosis mouse model (Apoe-/-Mmp10-/- mice) developed by Purroy et al. [17] by crossing Mmp10-/-; mice (B6.129P2-Mmp10tm1Jkmg) with Apoe-/-; mice (B6.129P2-Apoetm1Unc/J; Charles River Laboratories, L’Arbresle Cedex, France). Animals followed a standard chow diet throughout the experiment until sacrificed at 6, 10, 12 and 16 months.

All experiments were conducted according to the European Community guidelines for ethical animal care and use of laboratory animals (Directive 86/609) and were approved by the University of Navarra Animal Research Review Committee. 

#### 2.2.1. Quantitative Analysis at mRNA Level (qRT-PCR)

Aortas frozen in liquid nitrogen (n = 5/group) of 10 and 16 months of age were used to extract RNA using MagMAX-96 Total RNA Isolation Kit (ThermoFisher, Waltham, MA, USA). Reverse transcription was performed with 1 μg of total RNA, random primers and Moloney murine leukemia virus reverse transcriptase (ThermoFisher, Waltham, MA, USA). Real-time quantitative PCR was performed on an VIIA-7 sequence detector (ThermoFisher, Waltham, MA, USA) using TaqMan gene expression assays (IDT) for murine SDF1 (Mm.PT.58.32677664), CXCR4 (Mm.PT.58.41597935), and MMP12 (Mm.PT.58.31615472). Murine β-actin (Mm.PT.39a.22214843.g) was used to normalize results.

#### 2.2.2. Histological and Immunohistochemical Analysis

Immunohistochemistry was performed on 3 μm sections from the frozen aortas of 6 and 12 months of age mice. The slides were deparaffinized, and rehydrated, using antigen retrieval solution (Dakocytomation, ref S1699) diluted 1× at 95 °C for 20 min. Next, the samples were incubated with anti-CXCR4 (orb 74308, Biorbyt, dilution 1:50) and anti-MMP12 (orb36364, Biorbyt, dilution 1:100) antibodies for 24 h in moist chamber at 4 °C, washed appropriately and visualized by 3,3’dyaminobenzidine chromogen (DAB, Dako, ref K3468). Slides were then mounted with distyrene plasticizer and xylene mixture (DPX, VWR Chemicals).

Immunostained slides were subsequently scanned (Aperio ImageScope, Leica ByoSistems) and the percentage of positively stained area in total tissue area was quantified with Image-J software [21]. 

### 2.3. Statistical Analysis

Shapiro-Wilk test was used to evaluate normality. Normal values were expressed as mean and standard deviation (SD) and those without a normal distribution with median and interquartile range. MMP12 and CRP were logarithmic transformed to achieve a normal distribution. Association between two continuous variables was assessed by Pearson’s correlation test. Differences between two groups were analyzed by Student’s *t*- test or Welch-test as required by data distribution. Differences between more than two groups were assessed by one-way ANOVA followed by Bonferroni’s post-hoc test (two conditions). The Pearson’s χ^2^-test was used to compare frequency distribution of categorical variables. Univariate and multivariate logistic regression were used to stablish association between SDF1, MMP12, and CRP with clinical atherosclerosis manifestations, atherosclerotic plaques and other CV risk factors. The best discriminatory cut-off for SDF1 and CRP in association with clinal atherosclerosis manifestations was determined using the Youden’s index (Sensitivity−(1−specificity)) from the Receiver Operating Characteristic (ROC). A combined variable using SDF1 high/low and CRP high/low was created, to evaluate the association with clinical atherosclerosis manifestations. In the follow-up study, SDF1, MMP12 and CRP were stratified in terciles and a new variable combining them was created, considering high risk those who had SDF1, MMP12 and CRP in the highest tercile, low risk those with SDF1, MMP12 and CRP in the lowest tercile and medium risk any other combination. Time to the outcomes of interest was plotted using Kaplan–Meier curves for the combined variable, with statistical significance assessed using Cox-regression, controlled by age and sex. Statistical significance was set at *p* < 0.05 from two-sided test. Analyses were performed with STATA (version 12; StataCorp LP, College Station, TX, USA).

## 3. Results

### 3.1. Patients’ Characteristics 

This is a prospective study including 298 patients (71% males) with median age of 66 years (interquartile range (IQR) 59–76) that were followed-up for 33.6 months (1 day–57.5 months). Frequency of risk factors was as follows: hypertension 72%, DM 40%, dyslipidaemia 82% and active smoking 45.6%. As shown in Table 1 our cohort was then divided according to the presence or absence of clinical atherosclerosis: PAD (11.1%), CAD (19.5%), stroke/TIA (11.4%) and nephropathy (22.9%). Patients with clinical atherosclerosis manifestations (n = 120) were older (*p <* 0.001) and had higher abdominal perimeter (*p =* 0.04), hypertension (*p =* 0.003), DM (*p <* 0.001) and dyslipidaemia (*p =* 0.016) than the remaining group. Moreover, these patients presented reduced levels of total cholesterol (*p <* 0.001), low-density lipoprotein cholesterol (LDL-C) (*p <* 0.001) and high-density lipoprotein cholesterol (HDL-C) (*p <* 0.001), probably due to the effect of lipid lowering drugs, but increased levels of glycosylated haemoglobin (*p <* 0.001), triglycerides (*p =* 0.01) and creatinine (*p <* 0.001). Adherence to a Mediterranean diet was poorer in these patients (*p =* 0.05).

### 3.2. Association of Inflammatory Biomarkers with Cardiovascular Risk Factors

Univariate analysis showed an association between SDF1 levels and DM [OR (95%CI) = 0.63 (0.45–0.89), *p =* 0.008] and alcohol intake [OR (95%CI) = 0.82 (0.39–0.97), *p =* 0.035]. Likewise, MMP12 was associated with DM [OR (95%CI) = 1.56 (1.13–2.15), *p =* 0.007] and hypertension [OR (95%CI) = 1.56 (1.12–2.17), *p =* 0.008] (Table 2). Moreover, higher levels of CRP were found in patients with hypertension [OR (95%CI) = 1.22 (1.04–1.41), *p =* 0.012], DM [OR (95%CI) = 1.34 (1.17–1.52, *p <* 0.001] and dyslipidaemia [OR (95%CI) = 1.4 (1.08–1.82), *p =* 0.012] (Table 2).

On the other hand, all inflammatory biomarkers correlated positively with creatinine, and negatively with HDL-C and PREDIMED score. In addition, we found a significant correlation between circulating levels of CRP and SDF1 (r = 0.42, *p <* 0.001) and CRP and MMP12 (r = 0.31 *p <* 0.001), although no correlation between SDF1 and MMP12 was observed (Table 3).

### 3.3. Inflammatory Biomarkers in Relation to Clinical Atherosclerosis Manifestations

Patients with clinical atherosclerosis showed increased levels of SDF1 (2.7 ± 0.9 ng/mL vs. 2.3 ± 0.5 ng/mL, *p <* 0.001), MMP12 (501 [282–768] pg/mL vs. 358.5 [241.5–553.5] pg/mL, *p =* 0.002) and CRP (0.24 [0.13–1] mg/dL vs. 0.12 [0.07–0.31] mg/dL, *p <* 0.001). In the multivariate analysis, after adjusting for age, sex, DM, hypertension, dyslipidaemia, smoking, obesity, alcohol intake, and PREDIMED score (Table 4), both SDF1 [OR (95%CI): 2.4 (1.5–3.9) *p <* 0.001] and CRP [OR (95%CI): 1.6 (1.3–2.0, *p <* 0.001)] remained independently associated with clinical atherosclerosis. However, the association of MMP-12 and clinical manifestations was lost in the multivariate model.

ROC curves rendered an area under the curve (AUC): 0.66 ± 0.04 (95%CI: (0.59–0.73) and AUC: 0.68 ± 0.03 (95%CI: 0.62–0.73), for SDF1 and CRP respectively. The SDF1 ROC curve for clinical atherosclerosis manifestations was significantly improved when CRP was included in the model [AUC (95%CI): 0.70 (0.62–0.73); *p =* 0.005]. The cut-off values for SDF1 and CRP (2.46 ng/mL and 0.24 mg/dL, respectively) showed a sensitivity of 60.2% and a specificity of 65.3% for SDF1 and sensitivity of 63.9% and specificity of 68.2% for CRP. In multivariate analysis, patients with higher levels of SDF1 (≥2.46 ng/mL) were 2 times more likely to have presented clinical atherosclerosis than patients with lower SDF1 levels [OR (95%CI): 2.3 (1.2–4.4), *p =* 0.012, Table 5]. A similar multivariate analysis for CRP rendered a non-significant association with previous clinical atherosclerosis (Table 5). Furthermore, using these cut-off values we stratified our cohort into 3 groups. Group 1: low SDF1 and low CRP, group 2: either high SDF1 or high CRP, and group 3: high SDF1 and high CRP (Table 5). According to this distribution, the association of inflammation with clinical atherosclerosis manifestations was increased when evaluating SDF1 and CRP as a combined variable, regardless of the tested model (Table 5). In fact, model 3, including all confounding factors, showed an OR of 2.8 (95%CI: 1.2–6.8; *p =* 0.022) for the combination of SDF1 and CRP with clinical atherosclerosis.

### 3.4. Association of Inflammatory Biomarkers with Atherosclerotic Plaques

A total of 234 patients presented atherosclerotic plaques at the time of the analysis, although no differences in CV risk factors or demographic characteristics were observed when patients with or without plaques were compared (Table 6).

Likewise, no association between the number of territories affected by atherosclerotic plaques and the levels of SDF1, MMP12 and CRP were observed. 

### 3.5. Follow-Up Analysis

Patients were followed up for 33.6 months (range 1 day–57.5 months) and 6 patients were lost in follow up (2%). We registered 22 new cardiovascular events and 31 deaths, being 51.6% directly linked to atherosclerosis, 12.9% associated with cancer, 16.1% related to infections, 6.4% related to haemorrhage and 12.9% due to unknown reasons. 

**Figure 1:**
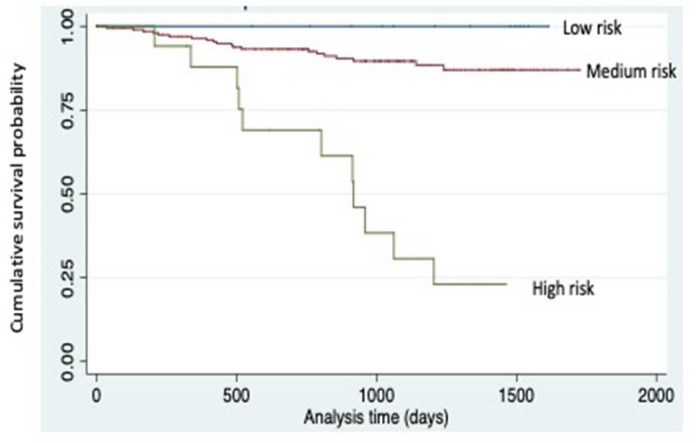
Survival according to combined risk variable. Combine risk variable stablished by the combination of SDF1, MMP12 and CRP. High risk: 3rd tercile SDF1 & MMP12 & CRP (n = 27), low risk: 1st tercile SDF1 & MMP12 & CRP (n = 15) and medium risk: any other combination (n = 256). Kaplan-Meier survival plots are unadjusted for covariates. A log-rank test of survival across risk levels of the combined variable is highly significant (*p <* 0.001).

Patients who died during follow up presented increased levels of SDF1 (3.1 (2.6–3.7) ng/mL vs. 2.4 (2.1–2.8) ng/mL, *p* = 0.001), MMP12 (612 (441–951) pg/mL vs. 384 (243–597) pg/mL, *p =* 0.001) and CRP (1.11 (0.35–2.10) mg/dL vs. 0.15 (0.07–0.33) mg/dL, *p <* 0.001). After adjusting by age and sex, those with increased levels of MMP12 or CRP presented an increased risk of death (HR = 3.2 (95%CI: 1.2–9.1), *p =* 0.026; and OR = 8.7 (95%CI: 2–37.9), *p =* 0.004; respectively). Additionally, those with SDF1, MMP12 and CRP in the highest tercile presented an increased risk of death, controlled by age and sex (HR = 3.2 (95%CI: 1.5–7), *p =* 0.004) with a Harrell’s-C index of 0.88 (Figure 1).

### 3.6. Aortic Expression of SDF1, CXCR4 and MMP12 

**Figure 2:**
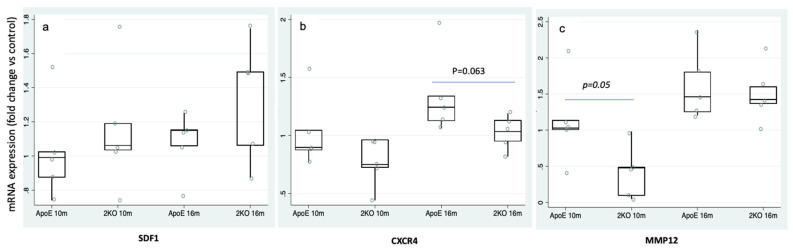
SDF1, CXCR4 and MMP12 expression in murine atherosclerotic aortas. (**a**) mRNA levels of SDF1 in aortas of Apoe-/- and Apoe-/-Mmp10-/- (2KO) mice at 10 and 16 months of age (n = 5/time point). (**b**) Aortic expression of CXCR4 was decreased in 2KO mice (n = 5/time point) vs. Apoe-/- at 16 months. (**c**) 2KO presented a reduction of MMP12 (n = 5/time point) vs. Apoe-/- at 10 months. * *p <* 0.05.

To further assess the role of inflammatory markers on the atherosclerotic process we analyzed the SDF1/CXCR4 signaling and assessed the SDF1, CXCR4 and MMP12 expression in the aortas of Apoe-/- and Apoe-/-Mmp10-/- (2KO) mice. SDF1 expression was similar at 10 and 16 months in both genotypes (Figure 2a), whereas CXCR4 was increased in Apoe-/-aortas at 16 months (1.4 ± 0.4-fold change vs. 1.0 ± 0.1-fold change, *p* = 0.063) vs. 2KO (Figure 2b). MMP12 in Apoe-/- was significantly increased earlier, at 10 months (0.4 ± 0.3 vs. 1.1 ± 0.6, *p* = 0.05), while at 16 months no differences between genotypes were found (Figure 2c). These results indicate that animals with higher atherosclerosis have increased aortic expression of CXCR4 and MMP12 genes.

**Figure 3:**
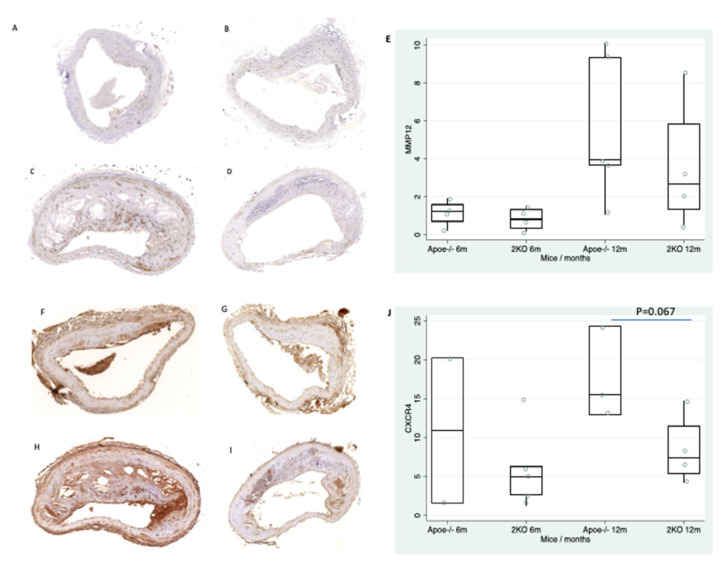
CXCR4 and MMP12 expression in murine atherosclerotic aortas. Expression of MMP12 in Apoe-/- mice (**A**) and 2KO mice (**B**) at 6 months. Expression of MMP12 in Apoe-/- mice (**C**) and 2KO mice (**D**) at 12 months. (**E**) Box plot showing the quantification of MMP12 expression in atherosclerotic plaques. Expression of CXCR4 in Apoe-/- mice (**F**) and 2KO mice (**G**) at 6 months. Expression of CXCR4 in Apoe-/- mice (**H**) and 2KO mice (**I**) at 12 months. (**J**) Box plot showing the quantification of CXCR4 expression in atherosclerotic plaques.

Next, we determined the CXCR4 and MMP12 protein expression in the aortas of Apoe-/- and 2KO mice at 6 and 12 months of age. Both CXCR4 and MMP12 increased with atherosclerotic progression (Figure 3E,J) but only CXCR4 expression showed a tendency at 12 months (17.6 ± 6 vs. 8.4 ± 4.5, *p =* 0.067) in Apoe-/- mice as compared with 2KO (Figure 3J).

## 4. Discussion

There is an unmet medical need to search for residual inflammatory risk in patients with atherosclerosis. We report herein that SDF1 may be a good marker of inflammation in CVD as it is significantly elevated in patients with clinical atherosclerosis. Additionally, higher levels of MMP12 and SDF1 were associated with increased risk of death after 33.6 months of follow up. Moreover, in experimental mice models of atherosclerosis (Apoe-/-) we confirmed increased expression of CXCR4 and MMP12 in aorta of accelerated atherosclerosis progression as compared with mice with delayed atherosclerosis (Apoe-/-Mmp10-/-), suggesting a pathogenic role for these inflammatory markers in atherosclerosis development.

SDF1 is an homeostatic chemokine playing a central role in the hematopoietic cell trafficking [22]. To date, SDF1 levels demonstrated to have prognosis impact for CV recurrence in patients with acute CV events; while we report the novel finding of increased levels in stable CVD, likely related with tissue remodeling and vascular dysfunction [23,24]. Our cohort presented elevated circulating SDF1 levels in patients with previous clinical atherosclerosis manifestations after adjusting for CV risk and other confounding factors.

MMP12 is produced by macrophages and it has been associated with plaque progression and instability [15]. Increased circulating levels of MMP12 have been reported in asymptomatic patients with high CV risk linked to carotid intima media thickness and cerebrovascular events during the follow up [25] as well as with the presence of CAD [26]. In our cohort, MMP12 was elevated in patients with previous atherosclerosis, although the association was lost after adjusting for confounding factors. 

In line with previous studies assessing the relevance of a multimarker approach for atherosclerotic risk assessment [27,28,29], our data indicate that high SDF-1 alone or even better when combined with high CRP levels were strongly associated with prevalent clinical atherosclerosis manifestations. Together, these data, emphasize that beyond the acute event, a residual inflammatory risk can be detected in these patients even if they are treated according to established guidelines. 

In addition, the prospective analysis showed that higher levels of SDF1, MMP12 and CRP were associated with death, rendering significatively elevated after adjusting for age and sex MMP12 and CRP. Additionally, the risk of death was increased in those with highest levels of all biomarkers, suggesting a potential link between increased residual inflammation and worse prognosis.

Since the underlying mechanism in CVD includes plaque formation, we analyzed the presence of atherosclerosis plaques and their association with the studied biomarkers. However, we did not find an association neither with the extension nor with the localization of the atherosclerotic plaques and any of the studied biomarkers. Previous studies reported association between CRP [30] and atherosclerosis, and MMP12 and atherosclerotic burden [15]. However, the different findings can be related to the heterogeneous characteristic of the populations.

Finally, to assess a possible pathogenic role of these proteins in atherosclerosis development we studied the aortic expression of MMP-12 and CXCR4, the main receptor of SDF1in an experimental model of atherosclerosis (Apoe-/- mice) as compared with a model of delayed atherosclerosis (Apoe-/-Mmp10-/- mice). We found that the aortic gene and protein expression of CXCR4 was increased in Apoe-/- mice compared to 2KO. The exact role of CXCR4/SDF1 in atherosclerosis is yet unknown. Some studies have reported that the expression of CXCR4 on macrophages was upregulated by pro-atherosclerotic factors [31,32], whereas other studies found a possible protective role of the CXCR4 in experimental atherosclerosis [33]. Moreover, Merckelbach et al. showed an increase of both CXCR4 and SDF1 in carotid atherosclerotic plaques, mainly in macrophages. Our results are in line with studies showing a proatherogenic role of CXCR4 and strengthened our previous findings of greater macrophage presence and larger atherosclerotic lesions in Apoe-/- [17,34]. Likewise, MMP-12 gene expression was significantly increased in Apoe-/- mice compared to 2KO. Interestingly, this MMP has been localized in macrophages associated with elastin and extracellular matrix degradation in atherosclerotic stroke patients [25].

There are some limitations of the study. The modest sample size and the low number of new events during a median follow up of 33 months are important methodological shortcomings. However, these results can be the basis for additional studies in larger cohorts to confirm these findings. The association between inflammatory markers and death during follow-up does not allow to establish a cause-effect relationship. Despite these limitations, the study shows that individuals with previous clinical atherosclerosis manifestations remain at increased CV risk with persistent elevations of inflammatory biomarkers associated with death during follow-up. Moreover, it is also remarkable that both SDF1 and MMP12 may be targeted by monoclonal antibodies, so they may represent new therapeutic targets in atherosclerotic disease.

## 5. Conclusions

In summary, the present study demonstrates that increased levels of SDF1, MMP12 and CRP identified a subgroup of patients with particularly high inflammatory cardiovascular and mortality risks. Multimarker model assessment and cost-effectiveness analysis are required for these markers in order to be integrated in daily clinical practice. In addition, the association of CXCR4 with atherosclerosis progression suggest that SDF1/CXCR4 signaling could be a possible atherosclerotic target.

## Figures and Tables

**Table 1:** Demographic characteristics of studied population according to the presence or absence of clinical atherosclerosis manifestations.

	No Clinical Atherosclerosis (n = 178)	Clinical Atherosclerosis (n = 120)	*p*
Sex (male)	123 (69.1)	88 (73.3)	0.43
Age (years)	61 (56–68)	74 (68–82)	<0.001
BMI (kg/m^2^)	28.7 (25.7–31.5)	28.6 (26.4–32.3)	0.39
Abdominal perimeter (cm)	102.2 ± 11.8	105.1 ± 11.5	0.04
Hypertension (yes)	117 (65.7)	98 (81.7)	0.003
Diabetes (yes)	46 (25.8)	73 (60.8)	<0.001
Dyslipidaemia (yes)	136 (76.4)	106 (88.3)	0.016
Systolic BP (mmHg)	136 ± 18	137 ± 20	0.75
Diastolic BP (mmHg)	84 ± 10	72 ± 16	<0.001
HbA1c (yes)	5.6 (5.4–5.9)	6 (5.5–6.8)	<0.001
Cholesterol (mg/dL)	184 (158–207)	154 (130–175)	<0.001
HDL-C (mg/dL)	57 (44–68)	45 (36.5–56)	<0.001
LDL-C (mg/dL)	107 (80–134)	79 (59–99)	<0.001
Triglycerides (mg/dL)	100 (72–136)	110 (81–193)	0.01
Creatinine (mg/dL)	0.9 (0.8–1)	1.16 (0.9–1.5)	<0.001
Smoking (yes)	32 (18)	15 (12.5)	0.2
Alcohol (yes)	38 (21.3)	14 (11.9)	0.031
PREDIMED score	9 (7–11)	8 (7–10)	0.05
SDF1 (ng/mL)	2.30 ± 0.49	2.72 ± 0.94	<0.001
MMP12 (pg/mL) ^a^	358.5 (241.5–553.5)	501 (282–768)	0.002
CRP (mg/dL) ^a^	0.12 (0.07–0.31)	0.24 (0.13–1)	<0.001

Continuous parametric variables are shown as mean (standard deviation), non-parametric as median (Interquartile range) and qualitative variables as number (%). ^a^ Logarithmically transformed variables. BMI: Body mass index, BP: Blood pressure, HbA1c: Glycosylated haemoglobin, HDL-C: High-density lipoprotein cholesterol, LDL-C: Low-density lipoprotein cholesterol, PREDIMED: PREvencion con DIeta MEDiterranea, SDF1: Stromal derived factor 1, MMP12: Matrix metalloproteinase 12, CRP: C-reactive protein.

**Table 2:** Association of inflammatory biomarkers with cardiovascular risk factors in univariate analysis.

	**SDF1 ** **OR (95%IC), *p***	**MMP12** **^a^** **OR (95%IC), *p***	**CRP** **^a^** **OR (95%IC), *p***
Hypertension	1.23 (0.85–1.76) 0.26	1.56 (1.12–2.17) 0.008	1.22 (1.04–1.41) 0.012
Diabetes mellitus	0.63 (0.45–0.89) 0.008	1.56 (1.13–2.15) 0.007	1.34 (1.17–1.52) <0.001
Dyslipidaemia	0.96 (0.64–1.44) 0.856	1.29 (0.9–1.86) 0.17	1.4 (1.08–1.82) 0.012
Obesity (BMI > 35)	0.82 (0.54–1.25) 0.36	1.35 (0.91–2) 0.13	1.09 (0.97–1.23) 0.14
Smoking (current)	0.54 (0.33–0.87) 0.54	0.75 (0.51–1.1) 0.14	0.9 (0.76–1.1) 0.29
Alcohol (>5/week)	0.82 (0.39–0.97) 0.035	0.84 (0.58–1.22) 0.37	0.81 (0.67–0.98) 0.028

^a^ Logarithmically transformed variables. BMI: Body mass index, SDF1: Stromal derived factor 1, MMP12: Matrix metalloproteinase 12, CRP: C-reactive protein.

**Table 3:** Correlation between cardiovascular risk factors and inflammatory biomarkers in patients with CV risk (n = 298) *.

	SDF1 (ng/mL)		MMP12 (pg/mL) ^a^		CRP (mg/dL) ^a^	
	R	P	R	P	R	P
Cholesterol (mg/dL)			0.12	0.03		
HDL-C (mg/dL)	−0.17	0.003	−0.26	<0.001	−0.42	<0.001
HbA1c (%)	−0.12	0.035	0.25	<0.001	0.28	<0.001
Diastolic BP (mmHg)	0.24	<0.001	−0.17	0.004	−0.49	<0.001
Waist (cm)			0.16	0.008	0.14	0.024
BMI			0.17	0.003	0.17	0.005
Creatinine (mg/dL)	0.27	<0.001	0.26	<0.001	0.53	<0.001
CRP (mg/dL) ^a^	0.42	<0.001	0.31	<0.001	-	-
SDF1 (ng/mL)	-	-	0.1	NS	-	-
PREDIMED score	−0.013	0.024	−0.13	0.03	−0.14	0.019

^a^ Logarithmically transformed variables. * Pearson correlation test. HDL-C: High- density lipoprotein cholesterol, LDL-C: Low-density lipoprotein cholesterol, HbA1c: Glycosylated haemoglobin, BP: Blood pressure, BMI: Body mass index, PREDIMED: PREvencion con DIeta MEDiterranea, CRP: C-reactive protein, SDF1: Stromal derived factor 1, MMP12: Matrix metalloproteinase 12.

**Table 4:** Multivariate logistic regression model to assess the association of inflammatory biomarkers and clinical atherosclerosis manifestations in patients with CV risk.

		OR (IC 95%)	*p*
SDF1	Crude	2.4 (1.6–3.5)	<0.001
	Model 1	1.6 (1.1–2.4)	0.018
	Model 2	2.3 (1.4–3.6)	<0.001
	Model 3	2.4 (1.5–3.9)	0.001
MMP12 ^a^	Crude	1.6 (1.2–2.3)	0.002
	Model 1	1.5 (1.1–2.2)	0.027
	Model 2	1.4 (0.9–2.0)	0.1
	Model 3	1.3 (0.9–2.3)	0.17
CRP ^a^	Crude	1.9 (1.6–2.3)	<0.001
	Model 1	1.6 (1.3–2.0)	<0.001
	Model 2	1.6 (1.3–1.9)	<0.001
	Model 3	1.6 (1.3–1.9)	<0.001

SDF1: Stromal derive factor, MMP12: Matrix metalloproteinase 12, CRP: C- reactive protein. ^a^ Logarithmically transformed variables. Model 1 includes age and sex, Model 2 includes hypertension, diabetes mellitus, dyslipidaemia and obesity, Model 3: Age, sex, hypertension, diabetes, dyslipidaemia, abdominal perimeter, smoking, alcohol, and PREDIMED score (PREvención con DIeta MEDiterranea).

**Table 5:** Association of clinical atherosclerosis manifestations and inflammatory biomarkers stratified by cut-off points for SDF1 and CRP alone and in combination.

**SDF1**				
	**<2.46 ng/mL** **OR (95% CI)**	**>2.46 ng/mL** **OR (95% CI)**		***p***
N	162	136		
Crude	1 (ref)	2.8 (1.8–4.6)		<0.001
Model 1	1 (ref)	1.6 (0.9–2.9)		0.088
Model 2	1 (ref)	2.4 (1.3–4.5)		0.006
Model 3	1 (ref)	2.3 (1.2–4.4)		0.012
**CRP**				
	**<0.24 mg/dL** **OR (95% CI)**	**>0.24 mg/dL** **OR (95% CI)**		***p***
N	184	114		
Crude	1 (ref)	2.2 (1.3–3.5)		0.002
Model 1	1 (ref)	1.3 (0.7–2.3)		0.4
Model 2	1 (ref)	1.2 (0.6–2.1)		0.62
Model 3	1 (ref)	1.3 (0.7–2.5)		0.37
**Categories of Combination Inflammatory Biomarker**				
	**G1 OR (95% CI)**	**G2 OR (95% CI)**	**G3 OR (95% CI)**	***p***
N	115	116	67	
Crude	1 (ref)	1.8 (1.0–3.1)	4.9 (2.5–9.3)	<0.001
Model 1	1 (ref)	1.2 (0.7–2.3)	2.0 (0.9–4.3)	0.09
Model 2	1 (ref)	1.5 (0.8–3.0)	2.5 (1.1–5.6)	0.032
Model 3	1 (ref)	1.7 (0.8–3.4)	2.8 (1.2–6.8)	0.022

SDF1: Stromal derived factor 1, CRP: C-Reactive protein, CV: Cardiovascular. Model 1 includes age and sex, Model 2 includes hypertension, diabetes mellitus, dyslipidaemia and abdominal perimeter, Model 3: Age, sex, hypertension, diabetes, dyslipidaemia, abdominal perimeter, smoking, alcohol, and PREDIMED score (PREvención con DIeta MEDiterranea). G1: SDF1 < 2.46 ng/mL and CRP < 0.24 mg/dL; G2: SDF1 > 2.46 ng/mL and CRP < 0.24 mg/dL cut-off or SDF1 < 2.46 ng/mL and CRP > 0.24 mg/dL; G3: SDF1 > 2.46 ng/mL and CRP > 0.24 mg/dL.

**Table 6:** Clinical and demographic characteristics of patients with CV risk according to the presence or absence of atherosclerotic plaques.

	Without Plaque (n = 64)	With Plaque (n = 234)	*p*
Sex (male)	36 (53)	175 (76)	0.32
Age (years)	63 (55–76)	67 (60–76)	0.15
BMI (kg/m2)	27.3 (25.7–30.4)	28.6 (26.1–32.0)	0.13
Abdominal perimeter (cm)	101.5 ± 13.6	104.0 ± 11.1	0.13
Hypertension (yes)	50 (73.5)	165 (71.7)	0.64
Diabetes (yes)	23 (33.8)	96 (41.7)	0.28
Dyslipidaemia (yes)	49 (72.1)	193 (83.9)	0.07
Systolic BP (mmHg)	135.6 ± 18.3	136.3 ± 19	0.77
Diastolic BP (mmHg)	77.6 ± 13.3	79.1 ± 13.9	0.43
HbA1c (%)	5.7 (5.4–6.3)	5.7 (5.5–6.3)	0.81
Cholesterol (mg/dL)	168 (147–203)	169 (148–198)	0.74
HDL-C (mg/dL)	55 (39–67)	50 (42–62)	0.71
LDL-C (mg/dL)	94 (70–127)	91 (72–120)	0.52
Triglycerides (mg/dL)	98 (70–130)	108 (78–149,5)	0.16
Creatinine (mg/dL)	1 (0.8–1.18)	1 (0.8–1)	0.8
Smoking (yes)	9 (13.2)	38 (16.5)	0.51
Alcohol (yes)	7 (10.3)	45 (6.5)	0.08
PREDIMED score	9 (8–10)	9 (7–10)	0.98
SDF1 (ng/mL)	2.5 ± 0.9	2.5 ± 0.7	0.85
MMP12 ^a^ (pg/mL)	381 (228–591)	424 (261–666)	0.11
CRP ^a^ (mg/dL)	0.18 (0.07–0.54)	0.17 (0.08–0.4)	0.16

Continuous parametric variables are shown as mean (standard deviation), non-parametric as median (Interquartile range) and qualitative variables as number (%). ^a^ Logarithmically transformed. BMI: Body mass index, BP: Blood pressure, HbA1c: Glycosylated haemoglobin, HDL-C: High-density lipoprotein cholesterol, LDL-C: Low-density lipoprotein cholesterol, PREDIMED: PREvencion con DIeta MEDiterranea, SDF1: Stromal derived factor 1, MMP12: Matrix metalloproteinase 12, CRP: C-reactive protein.

## Data Availability

The data presented in this study are available on request from the corresponding author. The data are not publicly available in accordance with consent provided by participants on the use of confidential data.
